# Clinical outcomes of radiation therapy for transgender and gender-expansive people with cancer

**DOI:** 10.3389/fonc.2023.1135400

**Published:** 2023-09-07

**Authors:** Arian Mansur, Abigail M. Kempf, Danielle S. Bitterman, Chirayu G. Patel, M Aiven Dyer, Daphne A. Haas-Kogan, Kevin X. Liu, Alicia C. Smart

**Affiliations:** ^1^ Department of Radiation Oncology, Brigham and Women’s Hospital, Boston, MA, United States; ^2^ Department of Radiation Oncology, Dana Farber Cancer Institute, Boston, MA, United States; ^3^ Department of Radiation Oncology, Massachusetts General Hospital, Boston, MA, United States; ^4^ Department of Radiation Oncology, Boston Children’s Hospital, Boston, MA, United States

**Keywords:** LGBTQ+, sexual and gender minorities, SGM, gender-affirming care, transgender, DE&I, patient-centered care

## Abstract

**Introduction:**

Approximately 1.6 million people in the US identify as transgender, many of whom undergo gender-affirming medical or surgical therapies. While transgender individuals are diagnosed with cancer at similar rates as those who are cisgender, the impacts of radiation therapy on outcomes of gender-affirming care in transgender, nonbinary, and gender-expansive people with cancer are understudied. We report on the experiences and outcomes of transgender and gender-expansive patients receiving radiation therapy for cancer treatment.

**Methods:**

This study is a multi-institutional retrospective review of patients evaluated from 2005-2019 identified as transgender or gender-expansive in the medical record and treated with radiation therapy.

**Results:**

We identified 23 patients who received radiation to 32 sites, including 12 (38%) to the brain, head, or neck, 8 (25%) to the thorax, and 7 (22%) to the pelvis. Seventeen patients (74%) received gender-affirming hormone therapy and 13 patients (57%) underwent gender-affirming surgery. Four patients had pelvic radiation before or after gender-affirming pelvic surgery, including two trans women who had pelvic radiation after vaginoplasty. Four patients had radiation to the chest or thorax and gender-affirming chest or breast surgery, including two trans men with breast cancer. Two pediatric patients developed hypopituitarism and hypogonadism secondary to radiation therapy and, as adults, changed their hormone replacement therapy to affirm their transgender identities.

**Discussion:**

Transgender people with cancer undergo radiation therapy for a wide range of cancers. Understanding their prior gender-affirming medical or surgical treatments and future gender affirmation goals may identify important considerations for their oncologic care.

## 1 Introduction

While there is a long history of transgender and gender-expansive individuals (1), increasing social acceptance and access to healthcare have contributed to a growing population that openly identifies as transgender. In the US, 0.6% of individuals identify as transgender (2), which approximates the prevalence of individuals with type I diabetes (3). In studies of transgender individuals in the US, 49% report undergoing gender-affirming hormone therapy and 25% have had gender-affirming surgery (4, 5). While data are limited, the overall incidence of cancer in transgender populations appears to be similar to rates in cisgender populations (6, 7). However, transgender people may be at increased risk for certain cancers, and there are unique treatment considerations for cancers that may be affected by gender-affirming hormone therapy or surgery (6, 7).

Additionally, many transgender, nonbinary, and gender-expansive people have had negative prior experiences with healthcare providers including being intentionally called by the wrong name or pronouns, experiencing verbal or physical abuse, and being refused care due to their transgender identity. In national surveys, 30% of transgender respondents reported delaying or avoiding medical care due to prior discrimination (8), and 24% of transgender cancer survivors reported that their oncology providers were not aware of their gender identity (9). Transgender and gender-expansive people experience healthcare disparities and may have unique needs (10). Meanwhile, a majority of oncologists report that they lack the experience or training to confidently meet the needs of transgender people with cancer (11, 12). A qualitative study of radiology and radiation oncology clinicians identified major gaps in training, knowledge, and confidence in caring for transgender people (13). Prior studies have reported outcomes of cancer treatment in cohorts of transgender patients (14, 15), but none have investigated the impact of radiation therapy (RT) on gender-affirming care.

## 2 Methods

This retrospective cohort of transgender and gender-expansive people with cancer treated at three institutions was identified, as previously reported, using keyword searches of each institution’s medical record and treatment planning system (14, 15). All three institutions currently have affiliated transgender health centers. Patients who did not receive RT were excluded. Survival analysis was performed using the Kaplan-Meier method with R version 4.0.3.

We use the terms trans woman, transgender woman, and transfeminine to refer to transgender individuals who were assigned male at birth and have female or feminine gender identities. The terms trans man, transgender man, and transmasculine refer to transgender individuals who were assigned female at birth and have male or masculine gender identities. The term nonbinary refers to individuals who have a gender identity that is not exclusively male or female. Gender-expansive is a term that broadly encompasses individuals with a wider or more flexible range of gender identity or expression.

## 3 Results

Twenty-three patients seen at our cancer center from 2005-2019 were identified in the medical record as transgender, nonbinary, or gender-expansive and received RT (**Table 1**). Most patients were documented as having a binary gender identity (e.g., transgender woman or transgender man) (17, 73%), were non-Hispanic white (21, 91%), and received their first course of RT as an adult (18, 78%). Ten patients (43%) were treated for hematologic cancers, 3 (13%) for primary brain tumors, and 10 (43%) for extracranial solid tumors. Five patients (22%) received multiple courses of RT, and 3 (13%) were treated with palliative intent. Of the 32 sites treated with RT, 12 (38%) included the brain, head, or neck, 8 (25%) included the thorax, and 7 (22%) included the pelvis.

**Table 1:** Clinical characteristics for 23 patients.

Characteristic	No. (%)
Sex assigned at birth	
Female	8 (35)
Male	15 (65)
Gender identity	
Woman	10 (43)
Man	7 (30)
Nonbinary	3 (13)
Genderqueer	1 (4)
Not specified	2 (9)
Race/ethnicity	
Non-Hispanic white	21 (91)
Hispanic white	1 (4)
Asian	1 (4)
Age at first RT	
<18 years	5 (22)
>18 years	18 (78)
Type of cancer	
Hematologic cancer	10 (43)
Lymphoma	7 (30)
Leukemia	2 (9)
Multiple myeloma	1 (4)
Brain tumor	3 (13)
Optic nerve glioma	1 (4)
Anaplastic astrocytoma	1 (4)
Non-germinomatous germ cell tumor of the pineal gland	1 (4)
Extracranial solid tumor	10 (43)
Breast	2 (9)
Lung	1 (4)
Sarcoma	1 (4)
Wilm’s tumor	1 (4)
Seminoma	1 (4)
Prostate	1 (4)
Rectal	1 (4)
Anal	2 (9)
No. of RT courses	
1	18 (78)
≥2	5 (22)
Treatment intent of first RT course	
Curative	20 (87)
Palliative	3 (13)
Site of RT	N = 32 courses
Brain	5 (16)
Head	3 (9)
Neck	2 (6)
Neck/Intrathoracic	2 (6)
Intrathoracic	2 (6)
Chest wall/breast/axilla	4 (13)
Abdomen	2 (6)
Lumbar spine	2 (6)
Pelvis	7 (22)
Extremity	1 (3)
Craniospinal	1 (3)
Total body irradiation	1 (3)

The median follow-up from first RT course for alive patients was 74 months (range 14-1442 months). Five patients died of disease; one died after discontinuing hemodialysis. The 5-year overall survival from first course of RT was 70% for all patients and 77% for patients treated with curative intent.

Only 3 patients (13%) had documentation of their pronouns (**Table 2**). Misgendering occurred in radiation oncology notes for 3 patients (13%). Examples included referring to a patient with he/him pronouns after documenting that the patient uses they/them pronouns, describing a patient who is a transgender woman as a man or male, and describing a patient who is transfeminine and nonbinary as a transgender man.

**Table 2:** Characteristics of gender-affirming therapy and medical documentation.

Characteristic	No. (%)
Any documented medical or surgical gender-affirming therapy prior to radiation	10 (43)
Documentation of patient’s pronouns	3 (13)
Misgendering in radiation oncology notes	
Yes	3 (13)
No	18 (78)
Radiation oncology notes unavailable	2 (9)
Gender-affirming hormone therapy	17 (74)
Gender-affirming surgery	13 (57)
Timing of gender-affirming surgery	
Before RT	7 (30)
After RT	6 (26)
No surgery	10 (43)
Sites of gender-affirming surgery	
Face/neck	2 (9)
Chest	11 (48)
Pelvis	8 (35)

Ten patients (43%) had documentation of gender-affirming therapy prior to radiation. The use of gender-affirming hormone therapy was reported for 17 patients (74%), and gender-affirming surgery for 13 patients (57%). Seven patients (54%) underwent gender-affirming surgery prior to RT and 6 patients (46%) had surgery after completing RT. Among the entire cohort, 2 patients (9%) had gender-affirming surgery involving the face or neck, 11 (48%) chest, and 8 (35%) pelvis. There were no grade 3 or greater complications at sites of gender-affirming surgery following RT.

### 3.1 Head and neck

Two patients underwent gender-affirming head and neck surgeries, including facial feminization surgery and tracheal shaving, but neither received RT to the brain, head, or neck. Five patients received intracranial RT to the mandible, and 4 to the neck. The histologies of the 3 intracranial tumors treated were optic nerve glioma, anaplastic astrocytoma, and non-germinomatous germ cell tumor of the pineal gland. There was no documentation about potential implications of head and neck RT for future gender-affirming surgery.

Two transfeminine patients received cranial RT as a child (one also received testicular RT) and developed panhypopituitarism and hypogonadism resulting in lack of expected male secondary sex characteristic development during puberty. Each patient was initially treated with testosterone therapy and subsequently switched to estrogen therapy. One patient’s parents were initially unsupportive of her gender transition. One patient later expressed interest in gender-affirming surgeries, including facial feminization.

Two pediatric patients developed meningiomas following cranial RT. One patient was on estrogen therapy when her meningioma was diagnosed. Her medical oncologist considered lowering her estrogen therapy as her testes were nonfunctional, and lower estrogen doses can produce similar results in patients with lower testosterone levels (16, 17).

### 3.2 Chest

Six patients in this cohort received curative intent RT to the chest or mediastinum—3 for lymphoma, 2 for breast cancer, and 1 for chronic lymphocytic leukemia (CLL). Eleven patients in this cohort underwent gender-affirming chest surgery; 7 were transmasculine and 4 were transfeminine. Two of these patients were trans men who received RT for breast cancer. Of the 2 patients treated for breast cancer, one trans man had a history of gender-affirming bilateral mastectomy and was later diagnosed with metastatic ER positive, PR weakly positive, HER2 negative breast cancer. His diagnosis was delayed by 1 year due to misdiagnosis of the mass as redistribution from prior mastectomy without obtaining imaging. Upon diagnosis, he underwent completion mastectomy utilizing the scar from his prior surgery and then received post-mastectomy RT. He had a history of gender-affirming hysterectomy and bilateral salpingo-oophorectomy and began testosterone therapy 10 years prior to diagnosis. A shared decision-making conversation with his medical oncologist concluded that he would continue testosterone therapy at half of his prior dose to balance his quality of life goals with the risk of testosterone promoting cancer growth. He was initially treated with letrozole and transitioned to tamoxifen due to progression.

Another trans man was diagnosed with an early-stage ER positive, PR positive, HER2 positive breast cancer and underwent bilateral mastectomy. The patient’s goals for gender affirmation were noted, among other factors, as a reason for choosing bilateral mastectomy. He subsequently was found to have a BRCA2 mutation and underwent prophylactic hysterectomy and salpingo-oophorectomy. He later developed metastatic recurrence, and while on trastuzumab/pertuzumab, he began gender-affirming testosterone therapy. Tamoxifen was added to reduce the risk of cancer growth, and his care was coordinated between his medical oncologist and primary care provider.

Three patients received RT for lymphoma (25.5-30 Gy), to the neck, mediastinum, or axilla. One transfeminine patient had breast augmentation surgery following RT to the neck, and her radiation oncologist documented that there was no significant RT dose to the breast or chest wall. Another trans woman had breast implants at the time of receiving RT to the axilla and breast for Hodgkin’s lymphoma. A trans man with a history of RT to the neck and mediastinum for Hodgkin’s lymphoma had gender-affirming bilateral mastectomies. His oncology team documented the need to determine appropriate cancer screening for his residual breast tissue in the context of mediastinal RT.

### 3.3 Pelvis

Eight patients underwent gender-affirming pelvic surgery, including hysterectomy and/or salpingo-oophorectomy in 2 patients, vaginoplasty or vulvoplasty in 4 patients, and orchiectomy alone in 2 patients.

Four patients, all transfeminine, had both RT and gender-affirming pelvic surgery. Two underwent gender-affirming orchiectomy and 2 had pelvic RT for rectal or anal cancers after vaginoplasty. Counseling and consent for a patient who underwent prostate RT and had plans for gender-affirming orchiectomy included discussion that orchiectomy would serve the function of lifelong androgen deprivation therapy for prostate cancer. The patient did not express interest in other genital surgeries but was advised that these would be more difficult following prostate RT.

Of the two patients who received pelvic RT after vaginoplasty, one patient continued vaginal dilator use three times per day during RT and the other patient had preexisting vaginal stenosis and received dilator training from radiation oncology nursing staff. Both patients reported symptoms of vaginal stenosis at follow up. One patient was planning for revision labiaplasty with skin grafting after completing cancer treatment. The radiation oncologist documented a conversation with her plastic surgeon regarding the timing and details of her planned surgery. RT dose to her external genitalia was minimized (mean 3.2 Gy) to reduce the risk of labiaplasty complications (18). The other patient was counseled that any future pelvic surgery plans should be discussed with her radiation oncologist due to the risk of wound healing complications after RT.

## 4 Discussion

Transgender patients receive RT for a wide range of cancers and have unique treatment needs. While some patients’ transgender identity may be identifiable based on their medication list or surgical history, others have not undergone medical or surgical therapies and information about their gender identity, name, and pronouns may be absent or incorrect in prior records. Patients may fear disclosing their transgender identity due to discrimination, which providers can alleviate by signaling that their clinic is welcoming of gender-expansive patients (9, 19). In this cohort, most patients did not have documented gender-affirming therapy prior to RT, and the study, including identification of the cohort, was limited by the availability and accuracy of medical records. While long term follow-up was available for many patients, documentation of the impacts of radiation on subsequent gender-affirming care was limited, possibly due to a lack of clinician awareness of potential impacts.

Clinic notes from existing providers inform a new provider’s initial understanding and expectations about a patient. Incorrectly documenting a patient’s gender or pronouns increases the likelihood of misgendering by other providers and propagation of misinformation throughout their record. Additionally, patient discovery of incorrect or unaffirming documentation may be detrimental to relationships with providers (20). Thus, we recommend asking every patient their name, pronouns, and gender identity and reflecting that language in notes (19). This strategy can also help providers avoid unintentionally using pejorative or outdated terminology.

Understanding a patient’s medical and surgical history and future gender affirmation goals is an important component of patient counseling and treatment decision-making. Radiation oncologists, in collaboration with the patient’s multidisciplinary oncologic and gender-affirming care teams, should engage their patients in shared decision-making. This approach should involve discussion of the uncertainties about the impacts of cancer treatment on gender-affirming care and enable patients to make informed decisions about their care. For example, two transmasculine patients in this series with ER positive breast cancers chose to initiate or continue testosterone therapy while undergoing cancer treatment, and one of these patients simultaneously began tamoxifen therapy to reduce the potential risk of ER-mediated growth of his cancer through aromatization of testosterone.

This shared decision-making framework should also be incorporated into RT consent discussions. The chest, genitals, and face are the most common sites of gender-affirming surgery (5, 21–23). For RT to a site of prior gender-affirming surgery, consent should include discussion of possible effects on the appearance or function of reconstructed tissues. Examples of potential risks include that transfeminine patients who have undergone vaginoplasty are at increased risk for vaginal stenosis following pelvic RT and should be counseled about vaginal dilator use (24). Two patients who had pelvic RT after vaginoplasty experienced vaginal stenosis, which for one patient was present prior to RT.

Transmasculine patients who undergo gender-affirming mastectomy or chest contouring surgery usually have residual breast tissue and should have shared decision-making conversations about breast cancer screening (25–27). Decreased physician awareness of breast cancer risk in transmasculine patients after gender-affirming mastectomy may have contributed to a delay in diagnosis for one patient who presented with a subcutaneous mass after chest surgery. Additionally, we expect that the risk of capsular contracture of breast augmentation implants in transfeminine people after RT is comparable to rates in cisgender women (28). One patient in this cohort underwent breast RT with an implant, receiving 30 Gy to the breast for Hodgkin’s lymphoma. Twelve years after RT, she underwent implant removal with bilateral capsular contracture, worse on the irradiated side.

We recommend asking all transgender patients about interest in future gender-affirming surgeries and counseling about how RT could impact those surgeries. If it will not delay oncologic care, surgical consultation prior to RT may improve patient understanding of the risks. In our cohort, discussion about planned labiaplasty enabled optimization of the RT plan for a patient with rectal cancer. Furthermore, transfeminine people should be counseled that pelvic radiation can preclude future full-depth vaginoplasty, although minimal-depth vaginoplasty, also known as labiaplasty or vulvoplasty, may remain an option (29, 30).

A patient’s gender affirmation goals may be directly relevant to oncologic treatment recommendations. A transfeminine patient with localized prostate cancer opted for orchiectomy for both gender affirmation and androgen deprivation therapy, and a trans man with BRCA2 mutated breast cancer chose bilateral mastectomy, hysterectomy, and salpingo-oophorectomy. However, assumptions about a transgender patient’s treatment preferences should be avoided. A transmasculine patient with breast cancer may be interested in mastectomy with or without reconstruction or may prefer breast conservation, and should be offered all appropriate options (27, 31).

The percentage of adolescents (age 13-17) who identify as transgender is almost three times higher than adults of age 25-64 (2). Providers should ask children and adolescents about their names and pronouns and refer to them accordingly. In some situations, a pediatric patient’s transgender identity may be directly relevant to their oncologic care. Two pre-adolescent patients developed treatment-induced pan-hypopituitarism, received hormone therapy to induce secondary sex characteristics of their sex assigned at birth, and subsequently switched to gender-affirming hormone therapy. While endocrine treatment in transgender adolescents or those with intersex conditions is beyond the scope of this discussion (32, 33), understanding the gender identity of a pediatric patient with radiation-induced hypogonadism may enable early referral to a gender care clinic. Additionally, all transgender cancer survivors may benefit from collaboration between their gender care providers and oncology team in determining appropriate cancer screening, surveillance, and management of late effects of cancer treatment.

The dearth of existing literature is a major limitation in oncologic care for transgender people. This study is the first, to our knowledge, to describe RT treatment and clinical outcomes for transgender people with cancer. Given the small size of the transgender and gender-expansive population, multi-institutional collaboration will be necessary to gain sufficient experience to inform RT recommendations for patients who have undergone or are considering gender-affirming medical or surgical therapies. Additionally, as the data from this study were extracted from review of medical records, this study does not incorporate patient-reported outcomes or perspectives. The involvement of the transgender and gender-expansive community in developing institutional practices and the design and conduct of clinical research is vital to ensure that such efforts are meeting patients’ needs, in addition to research reporting on patient-reported outcomes and experience measures. This is an essential future direction for our institution and many others to provide high quality care to transgender and gender-expansive people with cancer (34).

## 5 Conclusions

Transgender and gender-expansive patients are affected by a wide range of cancers and receive RT to sites of gender-affirming surgery, including the face, chest, and pelvis. Documentation of name, pronouns, and gender identity enables providers to correctly refer to patients and build respectful relationships. A patient’s history of gender-affirming surgery or future surgical plans may have important implications for RT consent and treatment planning. Late effects of oncologic treatment are best managed in collaboration with a patient’s multidisciplinary gender care team.

## Data availability statement

Data available on reasonable request to the corresponding authors due to privacy/ethical restrictions.

## Ethics statement

This retrospective study was approved by the Dana-Farber Cancer Institute Institutional Review Board. The study was conducted in accordance with the local legislation and institutional requirements. The institutional review board waived the requirement of written informed consent for participation from the participants or the participants’ legal guardians/next of kin because there is minimal risk from a retrospective chart review.

## Author contributions

MD, DH-K, KL, and AS contributed to the conception and design of the study. AM, AK, KL, and AS contributed to acquisition and analysis. All authors contributed to the interpretation of data. AM, AS and KL drafted the manuscript. All authors contributed to critical revision of the manuscript for important intellectual content.
